# [Corrigendum] Wentilactone A induces cell apoptosis by targeting AKR1C1 gene via the IGF-1R/IRS1/PI3K/AKT/Nrf2/FLIP/Caspase-3 signaling pathway in small cell lung cancer

**DOI:** 10.3892/ol.2026.15720

**Published:** 2026-06-22

**Authors:** Wenli Jiang, Linghong Meng, Guangming Xu, Cuiting Lv, Hongliang Wang, He Tian, Ruohua Chen, Binghua Jiao, Bingui Wang, Caiguo Huang

Oncol Lett 16: 6445–6457, 2018; DOI: 10.3892/ol.2018.9486

Subsequently to the publication of the above article, an interested reader drew to the authors’ attention that, in Fig. 4A on p. 6451, the fluorescence microscopic images chosen to show the results of the “NC (OE)” and “NC (KD)” experiments for the NCI-H1688 cell line, and the “KD” and “NC (KD)” experiments for the LTEPsm cell line, contained overlapping sections, such that these data, which were intended to show the results of differently performed experiments, had apparently been derived from the same original sources. In addition, a different reader raised concerns regarding the tumor sizes reported in Fig. 1E on p. 6447 and Fig. 6B on p. 6453, which appeared to be widely different from the sizes of the photos of the tumours portrayed in Figs. 1D and 6D, respectively, following their different treatments and after their having been harvested from the mice, and requested that the authors check their data/offer an explanation for this. The authors responded to this query by explaining that the tumor volume calculation formula L×w/2 was applied uniformly across all experimental groups and in all relevant figures (Fig. 1E, Fig. 6B and other supplementary figures), without any exceptions. The difference between the tumor volumes reported in their paper and those calculated by the interested reader arose from the fact that the tumor volumes in the study had been measured *in vivo*, rather than directly from excised tumors. This methodological difference led to discrepancies in the tumor volume measurements, but did not affect the core scientific conclusions of the study (the efficacy of WA, and the effects of AKR1C1 modulation on tumor growth, and response to WA treatment). Nevertheless, the authors acknowledged that they mistakenly placed the wrong statistical graph for Fig. 6B during the final figure assembly stage, and this error has been corrected in the revised version of Fig. 6 shown on a subsequent page (without any recalculation or modification of the raw data).

It was also noted by the Editorial Office, upon performing an independent analysis of the data in this paper, that there was potentially a further duplication of western blot control data, comparing Fig. 4B with Fig. 7; specifically, the control GAPDH blots featured in Fig. 7 for the NCI-H446 cell line closely resembled a trio of blots in the left-hand western blot featured in Fig. 4B, also for the NCI-H446 cell line.

The authors were able to consult their original data, and realized that certain of the data in Fig. 4A and B had inadvertently been assembled incorrectly. Along with the revised version of Fig. 6 (containing the correct graphical information for Fig. 6B), a revised version of Fig. 4, now showing the correct data for the “NC (KD)” experiment for the LTEPsm cell line and the “KD” and “NC (KD)” experiments for the NCI-H1688 cell line, and the control GAPDH blots in Fig. 4B for the NCI-H446 cell line, is shown on the next page. Note that the revisions made to these figures do not affect the overall conclusions reported in the paper. The authors are grateful to the Editor of *Oncology Letters* for granting them the opportunity to publish this Corrigendum, and all authors agree with its publication. Furthermore, the authors apologize to the readership for any inconvenience caused.

## Figures and Tables

**Figure 4.**
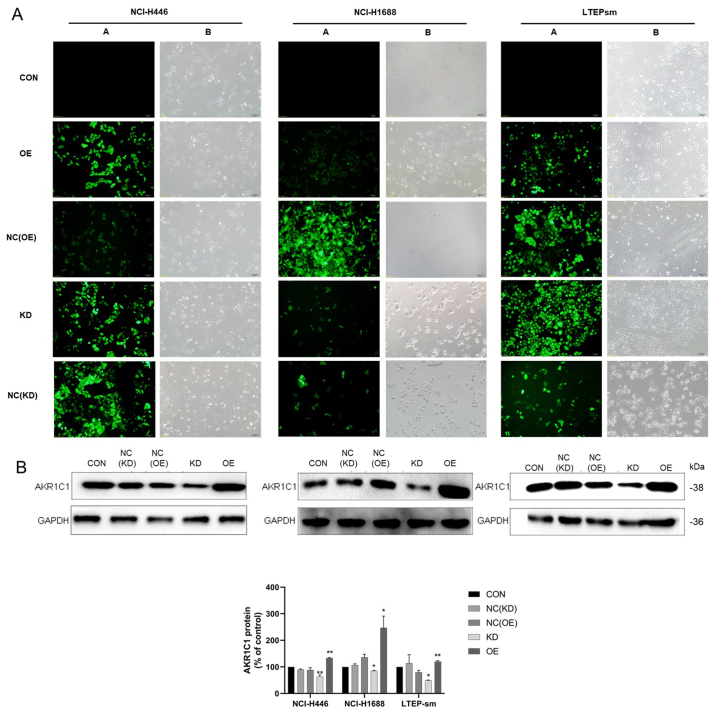
(A) Transfection efficiency was detected by fluorescence microscopy. (B) AKR1C1 protein level was detected by western blot analysis. AKR1C1 was significantly upregulated following transfection with AKR1C1 gene retroviral vector plasmid, and downregulated following transfection with lentiviral vector carrying sh-AKR1C1 plasmid. GAPDH was used as a reference. *P<0.05 and **P<0.01 vs. CON group. AKR1C1, aldo-keto reductase family 1 member C1; NC, control group; KD, lentiviral vector carrying sh-AKR1C1 plasmid; OE, transfected with AKR1C1 gene retroviral vector plasmid; CON, control group..

**Figure 6.**
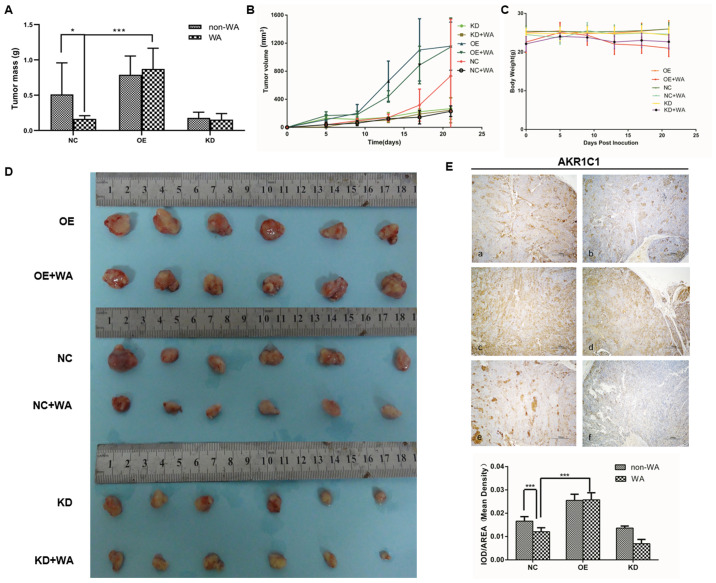
WA inhibited expression of AKR1C1 gene and tumor growth. There were six groups. OE group cells were infected with lentiviral vector LV5-AKR1C1; OE+WA group cells were infected with lentiviral vector LV5-AKR1C1 and WA treatment; NC group cells were non-WA-treatment; NC+WA group cells were WA treatment; KD group cells were infected with lentivirus particles carrying sh-AKR1C1; KD+WA group cells were infected with lentivirus particles carrying sh-AKR1C1 and WA treatment. (A) The weight of the metastatic tumors in each group. *P<0.05, ***P<0.001. (B) The subcutaneous tumor volume growth curve of WA treatment was depicted. (C) The body weight of the nude mice in each group. (D) Tumors were harvested following 20 days. (E) Immunohistochemical staining of (a) NC group, (b) NC+WA group, (c) OE group, (d) OE+WA group, (e) KD group and (f) KD+WA group. ***P<0.001. KD, lentiviral vector carrying sh-AKR1C1 plasmid; OE, transfected with AKR1C1 gene retroviral vector plasmid; AKR1C1, aldo-keto reductase family 1 member C1; NC, control group; WA, Wentilactone A; IOD/AREA, Integrated optical density per stained area.

